# Healthcare Professionals’ Perceptions of Lifestyle Medicine in Specialized Care: A Survey-Based Study in a Dutch Hospital

**DOI:** 10.1177/15598276251384634

**Published:** 2025-10-25

**Authors:** Catalina Paz Figueroa, Paula Bühring, W.M.Monique Verschuren, Marilyne Menassa, Oscar H. Franco, Lonneke van Leeuwen

**Affiliations:** 1 Department of Global Public Health & Bioethics, Julius Center for Health Science and Primary Care, 8124 University Medical Center Utrecht, Utrecht, Netherlands

**Keywords:** lifestyle medicine, perceptions, implementation, healthcare professionals, specialized care

## Abstract

Lifestyle Medicine (LM) offers promise for managing chronic diseases, yet its implementation in healthcare remains limited. Successful implementation requires understanding healthcare professionals’ (HCPs) perceptions, given their roles in advising patients and referring to lifestyle interventions. This study explores how HCPs at a Dutch academic hospital perceive LM. We conducted an anonymous online survey of physicians, nurses, and other HCPs in adult specialized care. We assessed perceptions of LM, how these varied across professional roles, and barriers and facilitators to implementation. We thematically analyzed free-text responses to identify factors influencing perceptions of lifestyle medicine. Of 303 participants, 14% were familiar with LM and 19% reported never discussing lifestyle with patients. However, 92% supported prioritizing lifestyle changes in healthcare and 69% prioritized implementing lifestyle programs in their hospital. 71% felt confident initiating lifestyle conversations, and 45% in promoting behavior change. Main barriers to discussing lifestyle included limited time (43%) and unclear referral pathways (38%). Thematic analysis generally revealed a positive perception of LM, but some questioned its suitability in specialized care. Most HCPs support LM, but LM’s role in specialized care requires clearer analysis and articulation. Greater awareness, practical support, and training seem key for successful implementation.

“While the importance of LM in healthcare is widely acknowledged, there is a need to further define the role of LM in specialized care...”

## Introduction

Lifestyle Medicine (LM) has emerged as a field dedicated to preventing, treating, and even reversing chronic diseases through evidence-based lifestyle interventions.^1^ LM addresses chronic diseases through six foundational pillars: a healthy diet, regular physical activity, restorative sleep, stress management, social connectivity, and the avoidance of risky substances like tobacco and alcohol.^2^

To date, efforts to implement LM interventions have primarily focused on primary care settings. General practitioners (GP) and primary care teams are usually well-positioned to address modifiable chronic disease risk factors and deliver sustained lifestyle interventions, given their regular patient contact and proximity to communities.^3-8^ In the Netherlands, GPs operate independently outside hospital settings and act as gatekeepers to specialized care.^9^ Specialized care includes hospitals and university medical centers, where healthcare professionals (HCPs) provide advanced diagnoses and treatments, deliver medical education, and conduct research.^10^ GPs focus on early prevention and management, while specialists typically treat patients with advanced disease or multiple risk factors. In acute care, specialists may encounter patients who are particularly motivated to improve their health due to life-threatening conditions.^11^ Similarly, during inpatient stays, they can address patients’ unhealthy lifestyle-related factors, which often persist throughout hospitalization.^12^ In outpatient clinics, they can discuss lifestyle risks contributing to patients’ illnesses and prognosis. All these patient–provider interactions can serve as valuable “teachable moments” for patients to promote lifestyle behavior change.^12^ Moreover, by aligning lifestyle recommendations with primary care providers, specialists can help maintain continuity of lifestyle-related care and support patients’ long-term health goals.^13,14^

Recently, UMC Utrecht and other major Dutch university medical centers implemented lifestyle front offices (LFOs).^15^ In these LFOs, health coaches use motivational conversations to support patients who are internally referred by HCPs. When appropriate, health coaches guide patients toward lifestyle interventions outside the hospital. The establishment of LFOs reflects a growing emphasis on integrating preventive care and LM into academic hospitals and specialist care. To maintain this momentum and ensure the sustained, long-term adoption of LM, HCPs serve as key stakeholders.^16^ They play a critical role in identifying patients’ needs for lifestyle interventions and incorporating lifestyle-focused care into clinical practice. However, HCPs are influenced by several factors when adopting LM practices.^17^ HCPs are particularly influenced by their own perceptions of their knowledge and skills in promoting behavior change. Additionally, they are influenced by their beliefs about whether promoting lifestyle change falls within their professional role. Specialist care is delivered within multidisciplinary teams, where physicians, nurses, and allied healthcare professionals each contribute distinct expertise and differ in the type and frequency of patient contact. Understanding these role-based differences is important because attitudes towards lifestyle care may vary with professional identity, training, and scope of practice. Moreover, understanding these differences can guide the tailoring of implementation strategies for different professional groups. In this study, we explore how HCPs at UMC Utrecht perceive LM in healthcare and how they perceive their role in delivering lifestyle-related care. We explore whether these perceptions differ across professional roles and assess the barriers and facilitators influencing LM implementation. By doing so, we aim to identify early opportunities for integrating LM into routine practice and improving referral behaviors in specialized care.

## Methods

### Study Design

We conducted a cross-sectional study using an online survey as a primary collection method for quantitative and qualitative data (through open-ended questions). This manuscript follows the STROBE (Strengthening the Reporting of Observational Studies in Epidemiology) guidelines for reporting cross-sectional studies.^18^ The study received ethical approval following institutional review at the University Medical Center Utrecht (UMC Utrecht).

### Study Context

This study was conducted at the UMC Utrecht, an academic hospital in the Netherlands that provides specialized healthcare services. Throughout this paper, the term “specialized care” refers to health services provided by specialized HCPs in hospitals and university medical centers (including both inpatient and outpatient departments). While other terms such as “secondary and tertiary care” are also used in the literature, “specialized care” is used here for consistency and readability. In the Dutch healthcare system, professionals working in medical (sub)specialties, such as physicians, nurses (including nurse specialists and specialized registered nurses), and allied health staff, are all considered healthcare professionals (HCPs). Allied health staff include physiotherapists, dietitians, midwives, occupational therapists, physchologists, and social workers. In specialized care, these HCPs may practice across various (sub)specialties, including surgical disciplines, internal medicine and its subspecialties, as well as inpatient and emergency care settings. Together, they contribute to multidisciplinary, specialized teams.

### Study Participants

HCPs were eligible for inclusion if they were employed in a clinical role involving direct patient contact at the UMC Utrecht. Exclusion criteria were: (1) working exclusively in pediatric care, or (2) employed exclusively in non-patient-facing clinical roles, such as those in education, research, diagnostics, or technical services (e.g., laboratory or imaging).

### Study Procedure

We recruited participants via a mailing list obtained from the Human Resources department. Eligibility was determined by reviewing the department and function data, to determine which met the inclusion criteria and did not meet the exclusion criteria. Out of approximately 12 000 hospital employees, 3960 were eligible. We sent them a single invitation email containing a link to the online survey. We used a range of additional recruitment strategies to encourage participation and reach those who might have been missed with the email list. These strategies included posters with QR codes placed around the hospital and text promotions on the intranet homepage, institutional newsletters, and social media platforms.

The survey was available for completion over 2 weeks, from January 23 to February 6, 2024. Informed consent was obtained online; completion of the survey was taken as consent, as outlined in the information letter. The survey was anonymous, and participants did not receive any form of compensation for participation.

### Survey Development

We designed the survey collaboratively with an interdisciplinary team from UMC Utrecht to minimize design bias and promote content validity. The team included experts in lifestyle medicine, epidemiology, preventive medicine, public health, and social research. We developed the survey guided by three key early-stage implementation outcome measures: acceptability, appropriateness, and adoption.^19^ Although we developed the survey without relying on pre-existing questionnaires, the survey shares similarities with published instruments measuring acceptability of lifestyle medicine or other healthcare interventions.^20,21^

### Survey Format

We delivered the survey in Dutch using Microsoft Forms. The survey included 2 sections. The first section included the questions for this study. The second section was designed to assess implementation outcomes of the LFO launched in 2022 at UMC Utrecht (these results are not reported here and will be submitted for publication elsewhere). The median time to complete both sections was approximately 9 min.

### Survey Items

The survey started with a screening question to confirm that participants met the inclusion criteria and did not meet any exclusion criteria. The questionnaire continued with twelve closed-ended questions and 2 open-ended questions (full questionnaire in Table S1). Question 1 identified the type of healthcare professional (physician, nurse, or other). To ensure anonymity, we did not collect any other identifying information. Question 2 assessed participants’ familiarity with LM. Regardless of their response, all participants received a brief explanation of LM before proceeding. The remaining questions addressed three early-stage implementation outcome measures: acceptability, appropriateness and adoption. Question 3 consisted of five statements assessing acceptability and appropriateness. Acceptability was operationalized as the perceived relevance and satisfaction with LM as part of healthcare. Appropriateness was operationalized as the perceived fit of LM within specialized care. Questions 4-12 assessed adoption, which was broadly operationalized as HCPs’ intentions to engage in LM practices, such as providing lifestyle advice and referring patients to structured lifestyle interventions. Specifically, questions 4-7 assessed HCPs’ current practices, questions 8-10 assessed perceived barriers and facilitators, and questions 11 and 12 assessed interest in further training. The closed-ended items included single-choice, multiple-choice, rating scale, and 5-point Likert scale formats.

The two open-ended questions invited participants to elaborate on their responses or share any other relevant thoughts, suggestions, or concerns. One open-ended question (Question OE1) was presented after the items on acceptability and appropriateness, the other one (Question OE2) was presented at the end of the questionnaire. In addition, Question 10 (about perceived barriers) included an “Other” option that allowed participants to provide a free-text response, which was incorporated into the thematic analysis. All questions were mandatory to complete, except for the open-ended questions and those that required a “yes” answer in the previous question (conditional follow-up questions).

## Data Analysis

We descriptively analyzed the results of closed-ended questions using Excel. Most questions are presented with frequency distributions of responses for each option (Questions 1, 2, 9, 11, 12). For items applying 5-point Likert scales (Questions 3, 4-8), we calculated the mean score and standard deviation (*SD*). Higher scores indicated greater confidence or greater agreement with the statements. For the question asking participants to rank all options in order of importance (Question 10), we calculated the mean ranking, with lower scores indicating higher perceived importance. For conditional follow-up questions, any skipped items were coded as “not applicable” rather than missing. Consequently, no item-level non-response error occurred, and no additional handling of missing data was required.

To examine statistical differences in responses by type of professional we used R studio (R version 4.4.0; 95% confidence intervals). Differences in response proportions were analyzed using Fisher’s Exact Test with simulated *P*-values for categories with expected frequencies <5, or Pearson’s Chi-squared test for categories with sufficient expected frequencies. For questions analyzed using mean score, we conducted a one-way ANOVA to assess statistically significant differences between groups. When the ANOVA indicated a significant effect, we performed post-hoc comparisons using Tukey’s Honestly Significant Difference (HSD) test to identify specific group differences.

We analyzed responses to open-ended questions using an inductive approach for thematic analysis in NVivo 14 software. The first author independently coded the free-text responses, beginning with open coding. This was followed by contrast coding to identify divergent or opposing views, as well as areas of consensus, regarding factors influencing perceptions of LM and implementation outcomes (acceptability, appropriateness, and adoption of LM). The resulting insights informed the subsequent organization of themes, which were then reviewed by a second author.

## Results

### Professional Background

**Figure 1.**
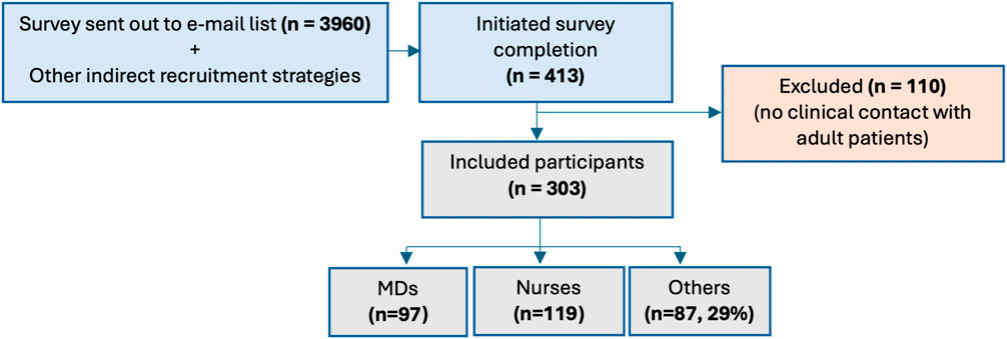
Flowchart of the sampling process. *Notes*: Abbreviations: n, number; MD, medical doctor.

A total of 413 individuals (10.4%) started the survey. Of these, 303 met the inclusion criteria and completed the questionnaire (response rate: 8%). Participants were roughly evenly distributed among physicians, nurses, and other non-medical healthcare professionals (Figure 1).

### Familiarity with Lifestyle Medicine

Among all participants, 14% were familiar with lifestyle medicine (LM), and 11% were unsure about their familiarity, with no significant differences across types of healthcare professional (Table S2).

### Acceptability and Appropriateness of Lifestyle Medicine

Table 1 presents participants’ agreement levels regarding the perceived importance of integrating lifestyle approaches in healthcare. Mean scores across the various dimensions of acceptability ranged from 3.8 to 4.3, indicating moderate to high acceptability. The highest level of agreement was with the statement emphasizing the importance of prioritizing lifestyle factors in healthcare (mean score: 4.3). Nurses expressed statistically significantly stronger support than physicians for implementing a lifestyle program in their hospital (*P* < 0.05). No other statistical differences between professional roles were found. Full response distributions by profession are provided in Table S3.

**Table 1.** Participants’ Mean Agreement Score to Different Statements.

Statement	Mean Score (*SD*)	Differences Across Roles
	N = 303	
“There should be a stronger focus on lifestyle factors within healthcare”	4.3 (0.7)	P = Nu = O
“Investing in lifestyle programs is an efficient way to reduce the pressure on healthcare”	4.1 (0.8)	P = Nu = O
“Investing in lifestyle programs is an efficient way of managing chronic diseases”	4.0 (0.8)	P = Nu = O
“Investing in lifestyle programs is an efficient way to reduce health inequalities”	3.8 (0.8)	P = Nu = O
“Implementing a lifestyle program for patients at the UMC Utrecht should be a priority”	3.8 (0.8)	*P < Nu P = O; Nu = O

*Notes*: Responses were measured on a 5-point Likert scale (1 = Strongly disagree, 5 = Strongly agree). Abbreviations: N, number of participants. SD, standard deviation; P, physicians; Nu, nurses; O, other healthcare professionals.

*Statistically significant differences between physicians and nurses (*P* < 0.05, based on ANOVA with Tukey post-hoc test).

### Adoption: Intentions and current practices

**Figure 2.**
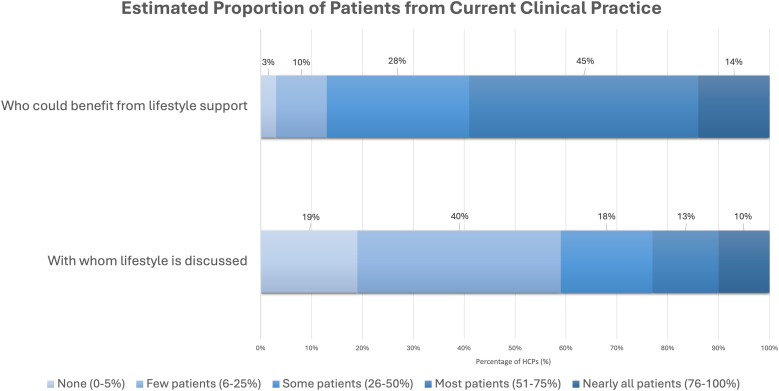
Healthcare Professionals’ Estimates of Patient Benefit From and Receipt of Lifestyle Support. *Notes*: Bars represent the percentage of HCPs who selected each response category, reflecting the proportion of their patients who (a) could benefit from lifestyle support or (b) are currently receiving such support through lifestyle discussions. Number of participants (n) = 303. Abbreviation: HCPs, healthcare professionals

Of all participants, 59% reported that over half of their patients could benefit from lifestyle behavior change support. An equal proportion (59%) reported currently discussing lifestyle with “none to few” of their patients (Figure 2). Physicians differed significantly from nurses and other HCPs in how often they discussed lifestyle with patients (*P* < 0.05). Most physicians (68%) reported discussing lifestyle with “few to some” (6%–50%) of their patients. In contrast, most nurses (68%) and other HCPs (54%) reported discussing lifestyle with “none to few” (0%–5%) of their patients. See Table S4 for detailed distributions.

**Figure 3.**
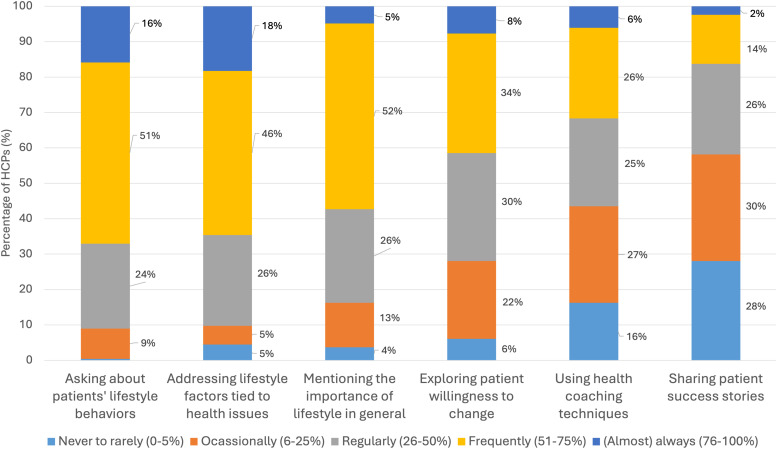
Frequency of Practices and Techniques for Addressing Lifestyle Topics With Patients Among Participants Who Discuss Lifestyle in Their Clinical Practice. *Notes*: Bars represent how frequently HCPs utilize various practices or techniques in interactions with patients. Number of participants (n) = 246. Abbreviation: HCPs, healthcare professionals.

Participants who discuss lifestyle with patients (n = 246) report diverse practices and techniques (Figure 3). More than half reported using three practices with over half of their patients: asking about patients’ lifestyle (67%), detailing specific lifestyle factors related to health issues (65%), and emphasizing the general importance of lifestyle (57%). They use other practices with over half of their patients less frequently: 42% asks about patients’ willingness to change, 32% use health coaching techniques (e.g., motivational interviewing), and 16% share patient success stories.

**Figure 4.**
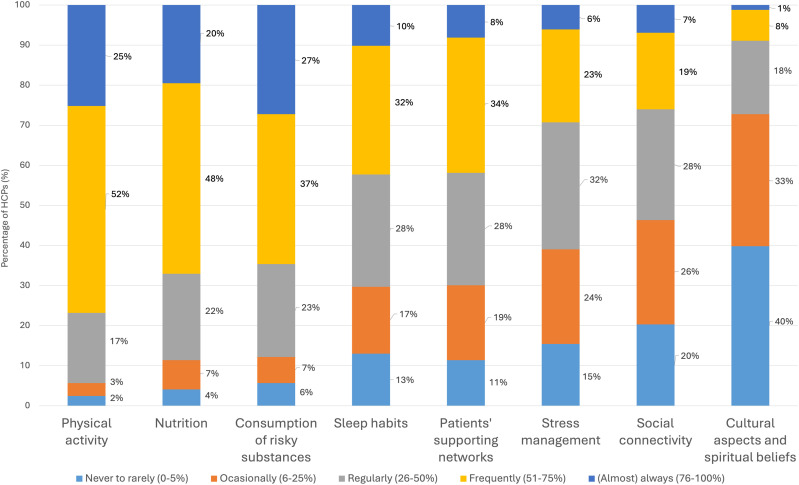
Frequency of Addressing Lifestyle Topics With Patients, Among Participants Who Discuss Lifestyle in Their Clinical Practice. *Notes*: Bars represent how frequently HCPs discuss lifestyle topics with patients (six pillars of Lifestyle Medicine, or two contextual factors influencing behavior change). Number of participants (n) = 246. Abbreviation: HCPs, healthcare professionals.

Participants who discuss lifestyle with patients (n = 246) also report varying frequencies in addressing specific lifestyle topics during consultations (Figure 4). More than half reported to discuss physical activity (77%), nutrition (68%), and consumption of risky substances (64%) with over half of their patients. They reported to address other topics with over half of their patients less frequently: 42% discuss patients’ support networks, 29% stress management, 26% social connectivity, and 9% cultural or spiritual aspects. Detailed distribution of responses by profession are available in Table S5.

### Adoption: Barriers and Facilitators

Table 2 presents the mean agreement scores on barriers and facilitators to adopting lifestyle practices. Overall, participants most strongly agreed with statements reflecting their confidence in opening lifestyle conversations with patients (3.7), their belief that lifestyle discussions increase their workload (3.7), and their confidence in their knowledge of lifestyle-related disease prevention (3.6). Nurses and other HCPs agreed significantly more than physicians with the statement expressing confidence in their ability to support patients in making lifestyle behavior changes (*P* < 0.05). In contrast, physicians agreed significantly more than nurses with the belief that discussing lifestyle is part of their job (*P* < 0.05). Participants showed the lowest mean agreement (2.3) with the idea that addressing patients’ lifestyle habits might lead them to adopt healthier behaviors themselves. Nurses and other HCPs agreed significantly more with this statement than physicians (*P* < 0.05). No significant differences across professional roles were found for other statements. Full response distributions by profession are provided in Table S6.

**Table 2.** Participants’ Mean Agreement Score to Different Statements.

Statement	Mean Score (SD)	Differences Across Roles
	N = 303	
“I am confident in my ability to open a conversation about lifestyle with patients.”	3.7 (0.8)	P = Nu = O
“(More) lifestyle discussions with my patients would increase my workload.”	3.7 (0.9)	P = Nu = O
“I am confident in my knowledge of the role of lifestyle in preventing and treating disease.”	3.6 (0.8)	P = Nu = O
“Discussing lifestyle with my patients is part of my job.”	3.5 (0.8)	*P > Nu; P = O; Nu = O
“My patients benefit from talking to me about lifestyle during my consultations.”	3.5 (0.9)	P = Nu = O
“I am confident in my ability to promote lifestyle behavior change in patients.”	3.3 (0.9)	*P < Nu, O
“I know how to refer my patients who could benefit from lifestyle changes.”	2.9 (0.9)	P = Nu = O
“If I paid more attention to lifestyle in my patients, I would have a healthier lifestyle myself.”	2.3 (1.0)	*P < Nu, O

*Notes:* Responses were measured on a 5-point Likert scale (1 = Strongly disagree, 5 = Strongly agree). Abbreviations: N, number of participants; SD, Standard Deviation; P, physicians; Nu, nurses; O, other healthcare professionals.

*Statistically significant differences among professional roles (*P* < 0.05, based on ANOVA with Tukey post-hoc test).

The most frequent barrier was a lack of time to discuss lifestyle with patients during clinical encounters (43%). Other common barriers were insufficient knowledge of patient referral processes for lifestyle programs (38%), perceived patients’ disinterest (33%), and patients’ socio-economic limitations (30%). A summary of all endorsed barriers is presented in Table 3.

**Table 3.** Percentage of Participants Endorsing Various Barriers in Lifestyle Discussion During Clinical Encounters.

Barriers	Total % (CI)
	N = 303
1) Lack of time	42.6 (42.3, 42.9)
2) Lack of knowledge on how or where to refer patients	37.6 (37.3, 37.9)
3) Perceived patients’ disinterest	33.3 (33.0, 33.6)
4) Perceived patients’ socio-economic barriers	30.3 (30.1, 30.7)
5) Other barriers	20.1 (19.9, 20.4)
6) Lack of confidence in own lifestyle medicine knowledge and/or skills	18.5 (18.2, 18.7)
7) Not considering it part of their job	15.1 (14.9, 15.4)
8) Disbelief in the efficacy of discussing lifestyle with patients	7.3 (7.1, 7.4)

*Notes:* Participants could select all options that applied. Abbreviations: N, number of participants; CI, 95% confidence interval.

Two factors emerged as the most important facilitators for referring patients to lifestyle programs. These were a simple and straightforward referral process, and a clear protocol with well-defined information about the available lifestyle program(s). Hearing success stories from patients who attended these programs ranked third in importance. Table 4 summarizes the mean rankings of all identified facilitators.

**Table 4.** Participants’ Ranking of Facilitators for Referring Patients to Lifestyle Programs.

Facilitators	Mean ranking
	N = 303
1) A simple and easy referral process	2.7
2) Clear protocol and information about the program(s)	2.8
3) Knowing patients’ success stories	3.6
4) Possibility to directly collaborate with the program(s)	3.9
5) It being recommend by colleagues	4.3
6) Receiving a budget/incentive for lifestyle training opportunities	4.9
7) It being required by the manager/department/institution	5.8

*Notes*: Participants ranked all options in order of importance. Lower scores indicate higher perceived importance. Abbreviation: N, number of participants.

### Adoption: Interest in Lifestyle Medicine Education

Finally, 51% of participants expressed interest in expanding their LM knowledge, 31% indicated “maybe,” and 18% expressed no interest, with no statistically significant differences among professional roles (*P =* 0.066). Among those (potentially) interested (n = 250), they favored the following learning methods: institutional e-learning modules (52%), departmental workshops and seminars (46%), and symposia and conferences (36%). Less than a quarter of participants selected other options, such as online webinars (23%), mentorship by a lifestyle practitioner (20%), formal training in the field (19%), podcasts (17%), institutional newsletters (16%), and free subscriptions to national LM resources (15%). Detailed selection distribution is available in Table S7.

### Thematic Analysis

Of the 303 participants, 201 (66%) provided a total of 312 free-text comments. After excluding 18 non-substantive responses, 294 comments were subjected to analysis. Twenty comments were unclassifiable under any theme: 13 contained neutral or unclear messages, 7 were positive towards the survey topics, and none were negative. These 20 comments lacked specific reasons, such as “I don’t think it should be a priority, but it should be important” or “As far as I’m concerned, a healthy lifestyle is very important.” Therefore, we excluded them from the thematic analysis. The remaining 274 comments reflected participants’ perceptions of LM. Some comments were lengthy and addressed multiple subthemes, resulting in a total of 335 classified comments or codes. These data informed fourteen themes, capturing both areas of consensus and divergent views that may influence perceptions and implementation of lifestyle medicine. A summary of the identified themes is presented in Table 5.

**Table 5.** Fourteen Themes Summarizing Healthcare Professionals’ Perceptions of Lifestyle Medicine (LM).

	Theme	Summary of key points
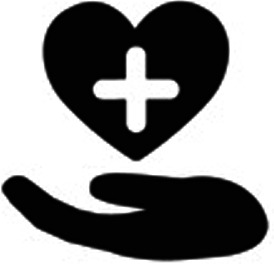	Understanding of lifestyle medicine (n = 63)	Mixed perceptions on LM scope, from a complementary to a foundational discipline in healthcare. LM was often equated with primary prevention, though its roles in disease treatment and mental health were also highlighted.
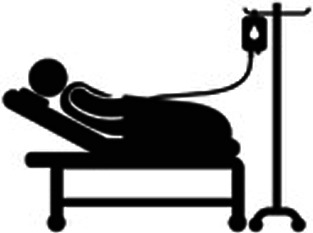	Medical disciplines and provider roles (n = 49)	Mixed perceptions on the relevance of lifestyle discussions. Some providers express high-acuity settings (e.g., ER, OR, ICU) limit opportunities for lifestyle discussions, while others see every patient encounter as a chance to discuss lifestyle.
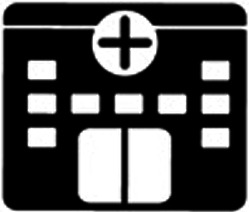	Role of academic hospitals (n = 45)	Mixed perceptions on integrating LM in academic hospitals, with some seeing it as primarily for primary care and others advocating for its inclusion across all healthcare levels. The debate centered on whether academic hospitals should implement lifestyle programs directly or serve mainly as referral points to community programs.
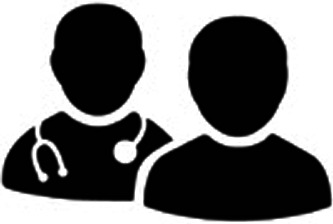	Patient–provider interactions (n = 34)	Mixed perceptions about patients’ degree of lifestyle knowledge and motivation for behavior change. Consensus on the need to improve provider skills for promoting behavior change. Mixed experiences with lifestyle discussions, with some providers describing them as stimulating and rewarding, and others expressing boredom or disengagement.
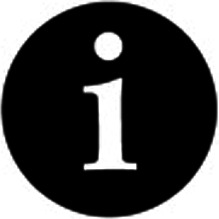	Visibility and accessibility (n = 25)	Consensus on the need to raise awareness of LM and the importance of advertising existing lifestyle programs. Clear referral routes and insurance coverage details seen as key.
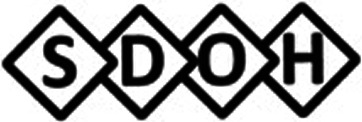	Social determinants of health (n = 22)	Mixed perceptions on LM’s capacity to reduce inequities. Consensus that effective interventions must address health literacy, affordability, and access to healthy foods.
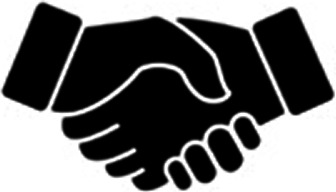	Accountability (n = 18)	Mixed perceptions on who bears responsibility for health promotion, ranging from individual to governmental responsibility. Some argue LM is better suited to public health and policy efforts than to clinical care.
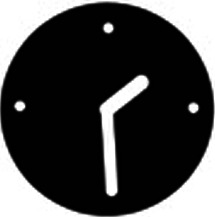	Time paradox (n = 17)	Consensus on the tension between short consultations and the need to provide long-term LM support. Proposed solutions include referrals to dedicated programs, adding LM specialists, longer appointments.
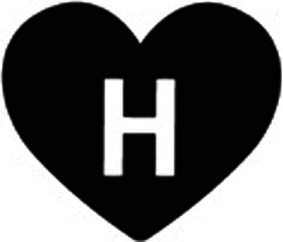	Healthy hospital (n = 16)	Consensus on transforming hospitals into models of health-promotion for staff and patients through healthier work and healing environments. One participant expressed hope that this transformation could raise HCPs’ awareness of their own lifestyles.
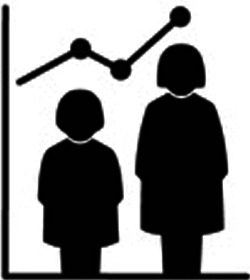	Demographics (n = 11)	Some participants expressed concerns about implementing LM in adult hospitals (“too late”). Proposed solutions include to target children, young parents, and relatives to achieve better long-term outcomes.
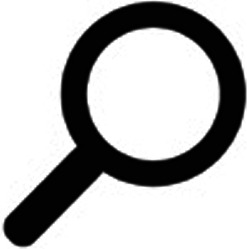	Scientific evidence (n = 10)	Mixed perceptions: Some call for more rigorous research before LM adoption, while others argue evidence is sufficient and stress the need for effective implementation.
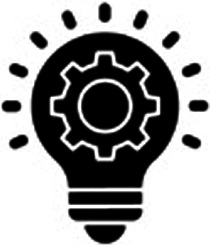	Building on existing knowledge (n = 9)	Some participants emphasized drawing on local expertise and integrating with existing practices to prevent duplication of efforts and minimize top-down decision-making.
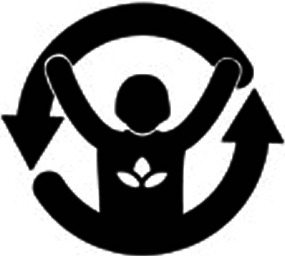	Definition of health (n = 9)	Consensus on LM being more effective when adopting a holistic view of health and well-being. One participant warned against chasing unrealistic health ideals that may reduce enjoyment.
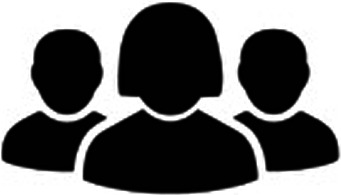	Multidisciplinary collaboration (n = 7)	Consensus on the role of non-physician HCPs in LM delivery, with nurses seen as well-positioned due to their close patient contact.

*Notes*: Themes identified in comments expressed by 201 participants (66%), including points of consensus and divergence. Abbreviation: N, number of participants mentioning the theme. ER, emergency room; OR, operating room; ICU, intensive care unit.

## Discussion

To the best of our knowledge, this study is among the first to broadly examine how healthcare professionals (HCPs) perceive Lifestyle Medicine (LM) within a specialized academic hospital. Most participants were unfamiliar with LM as a distinct discipline. Still, many supported prioritizing LM in healthcare and frequently equated LM with primary prevention. However, it is important to recognize that LM extends beyond primary prevention, as it also employs evidence-based interventions for managing and reversing chronic diseases.^2^ This broad understanding of prevention seems fundamental. Our qualitative findings suggest that when participants perceived LM either as solely a form of primary prevention or as a governmental/public health responsibility, they were less likely to perceive it as appropriate in academic hospitals treating adult patients. Despite this, many participants in this survey view lifestyle discussions as part of their professional role and believed that patients benefit from such conversations. This is an important finding, as previous research has shown that such perceptions strongly influence the implementation of lifestyle interventions and may support greater adoption of lifestyle practices in routine care.^17^

The identified barriers to adoption were consistent with those found in previous studies, with lack of time being the most commonly reported.^17,22^ To address time constraints as a barrier, hospitals could work on integrating brief lifestyle interventions (BLIs). BLIs, taking only 30 seconds to several minutes, can initiate meaningful changes in patient motivation for behavior change.^23-25^ Although most research on BLIs has focused on primary care, some examples in specialized care include cardiologists and physiotherapists providing brief lifestyle advice during cardiac rehabilitation,^26^ and emergency departments delivering brief smoking cessation interventions during patients’ visit.^27^ Hospitals could also integrate group consultations, which can provide both lifestyle guidance and peer support to multiple patients sharing similar chronic conditions.^28,29^

In addition to time constraints, specialized care settings present unique challenges when supporting patients to make lifestyle behavior changes. HCPs in areas such as intensive care, oncology, or palliative care often work with highly complex patient populations, where other priorities may come first. Clinical guidelines often lack practical recommendations or evidence-based lifestyle support tailored to these specific patient populations.^30^ To address this gap and strengthen LM implementation, hospitals could adopt clear, evidence-based lifestyle guidelines and protocols for clinical teams managing complex chronic illnesses. For example, oncology teams could benefit from guidance on supporting breast cancer patients to engage in physical activity.^31,32^ Similarly, palliative care teams could be equipped to implement balanced lifestyle interventions aimed at improving quality of life for patients with advanced or terminal diseases.^33^ Furthermore, hospitals could strengthen implementation by identifying and evaluating existing lifestyle program offerings and ensuring they are visible, accessible, and supported by a simple referral process.^34^

HCPs also reported barriers related to their perceptions of patients. In particular, the perceived lack of patient interest in lifestyle support may represent a significant myth that needs debunking.^17^ Evidence from primary care studies has shown that patients often seek support from their physicians to make lifestyle changes and report greater motivation to lose weight than their physicians typically recognize.^35,36^ In addition, HCPs expressed mixed perceptions on whether their patients have sufficient knowledge about healthy lifestyle habits. Despite these mixed perceptions, participants in this survey expressed a common recognition of the importance of developing skills to support lifestyle behavior change, and most HCPs expressed interest in learning more about LM. These attitudes suggest a strong foundation on which to build broader adoption of LM practices in specialized care settings. Tailored training in strategies for efficient behavior change can equip all HCPs across diverse clinical contexts with the knowledge and skills needed to motivate patients to adopt lifestyle changes.^37,38^

Participants in this survey highlighted multidisciplinary collaboration and empowering non-medical HCPs as opportunities to increase the adoption of lifestyle practices, which aligns with emerging multidisciplinary lifestyle approaches in primary care.^39,40^ Nurses strongly supported institutionalizing lifestyle programs, and the thematic analysis revealed interest and support for expanding nurses’ responsibilities in delivering lifestyle care. However, many nurses did not perceive lifestyle discussion as part of their role and engage in lifestyle discussions less frequently than physicians. At the same time, nurses and other non-medical HCPs reported greater confidence than physicians in discussing lifestyle with patients. According to a recent umbrella review, nurse-led consultations are at least as effective as physician-led consultations in supporting patients with complex health conditions.^41^ In addition, nurse-led consultations are potentially superior in improving health behaviors and increasing patient satisfaction. These findings support the notion that the role of nurses in implementing lifestyle interventions should be strengthened. They also highlight the need to support role-specific training and to explore collaborative care models in academic hospitals, such as nurse-led clinics or interprofessional teams, where non-physician professionals take leading roles in delivering lifestyle care.

In terms of current practices, HCPs in our study reported addressing more frequently physical activity, nutrition, and avoidance of toxic substances, which was in line with previous literature.^30,42^ In contrast, most HCPs reported not routinely addressing sleep habits, patients’ supporting networks, stress management, or social connectivity. Moreover, they reported rarely inquiring about cultural aspects influencing patient lifestyle behaviors. These omissions may be particularly relevant in the context of a country like the Netherlands,^10^ where immigration has risen significantly in recent years, with over 300 000 new arrivals annually. Patients with a migration background may have diverse cultural norms, beliefs, or barriers related to health behaviors, which can affect the effectiveness of lifestyle advice.^43^

Many HCPs recognized that addressing social determinants of health (SDoH) and prioritizing populations with fewer resources is necessary for implementing LM and reducing health inequities. The Dutch National Institute for Public Health and the Environment^44^ also acknowledges this issue, as do some of the largest LM organizations worldwide.^45,46^ One promising solution to help HCPs address SDoH for lifestyle behavior change could be integrating social prescribing models. Social prescribing shifts the responsibility of navigating non-medical needs to dedicated roles, such as health coaches or link workers.^47,48^ Lifestyle Front Offices (LFOs) could be an example of this model in practice, connecting patients to community resources and social support services.^15^

Finally, there is a clear need for dedicated LM implementation research in specialized care. While inpatient initiatives can initiate positive behavioral changes, sustainable lifestyle modifications require long-term support that extends beyond the hospital environment. The Dutch Association of Physicians and Lifestyle (*Arts en Leefstijl*^49^) has set the mission of achieving “a healthier Netherlands through the application of Lifestyle Medicine,” aiming to permanently integrate LM into regular healthcare by 2030. The “Coalition Lifestyle In Healthcare” (*Coalitie Leefstijl in de Zorg*) aims to develop knowledge for establishing lifestyle as a regular part of healthcare.^50^ To support these goals, more research is needed to investigate whether LM programs in academic hospitals are as effective as those implemented in primary care. Hospitals should focus on developing cost-effective, viable business models to implement effective LM interventions.^51^ Moreover, hospitals should focus on promoting a supportive and collaborative workplace culture: one characterized by interdisciplinary teamwork, role modeling by colleagues, and a health-promoting physical environment, which could enhance LM adoption, patient recovery, and reduce stress on clinical staff.^52,53^

## Strengths and Limitations

This study presents several strengths, including its pioneering exploration of Dutch HCPs’ perceptions in specialized care. This research establishes a foundational framework for future research and for guiding LM implementation plans. The cross-sectional design enabled efficient data collection, while the use of mixed methods allowed for the capture of diverse views. The anonymity of participants helped mitigate potential social desirability bias. Self-assessments of skills and knowledge may be subject to bias, as participants could overestimate or underestimate their abilities. However, perceived abilities remain important because they influence HCPs’ behavior and decisions, irrespective of their skill level.

In terms of limitations, the use of a convenience sampling method introduces volunteer bias, as HCPs with strong views toward LM (positive or negative) may have been more likely to participate. Given the modest response rate, it is uncertain whether the sample fully represents the broader population of HCPs at UMC Utrecht. The findings may be skewed and not fully reflect the attitudes and practices of those who are less engaged or indifferent to LM. Lastly, we could not apply weighting or propensity scoring to improve the representativeness of the sample because demographic variables other than professional role were not collected, in order to preserve anonymity and encourage candid responses. Together, this may limit the generalizability of the results. However, a strength of the study is that professional roles were evenly represented, suggesting that the perspectives captured were not dominated by a single subgroup of HCPs.

Additionally, some questionnaire items may have been poorly phrased or ambiguously worded, leading to misinterpretations or misunderstandings. The lack of a survey pre-test increases the risk of including misleading questions, which may compromise the validity of responses. We proceeded without pre-testing to enable timely deployment and inform ongoing implementation efforts. Nevertheless, the survey was developed with expert input and aligned with previously published instruments, which we expect to mitigate, though not eliminate, these risks.

These limitations should be considered when interpreting the study’s findings and when making recommendations based on these data. To gain a more comprehensive understanding of HCPs’ perceptions of LM, future studies should consider using complementary methods, such as interviews or focus groups, to capture more nuanced and in-depth insights.

## Conclusions

Generally, healthcare professionals at UMC Utrecht support the role of Lifestyle Medicine (LM) in managing chronic diseases and alleviating the strain on the healthcare system, primarily recognizing its preventive role. However, some concerns were raised about its appropriateness in academic hospitals. While the importance of LM in healthcare is widely acknowledged, there is a need to further define the role of LM in specialized care and to address barriers to its implementation. Key strategies for improving early implementation outcomes include increasing awareness through targeted communication campaigns, establishing clear protocols and referral pathways for available lifestyle programs, enhancing knowledge through tailored LM training, encouraging multidisciplinary collaboration, and cultivating a healthy hospital environment that sets a positive example. By addressing these areas, hospitals can better position themselves to implement effective LM initiatives that benefit both patients and healthcare professionals.

## Supplemental Material


Supplemental material - Healthcare Professionals’ Perceptions of Lifestyle Medicine in Specialized Care: A Survey-Based Study in a Dutch Hospital
Supplemental material for Healthcare Professionals’ Perceptions of Lifestyle Medicine in Specialized Care: A Survey-Based Study in a Dutch Hospital by Catalina Paz Figueroa, Paula Buhring, Monique W.M. Verschuren, Marilyne Menassa, Oscar H. Franco and Lonneke van Leeuwen in American Journal of Lifestyle Medicine

## Data Availability

The datasets generated during and/or analyzed during this study are available from the corresponding author upon reasonable request. In such cases, the suitability of the data for the intended use will be assessed, and a data-sharing agreement will be established if appropriate.*
