# Supplementary material for: Healthcare Professionals’ Perceptions of Lifestyle Medicine in Specialized Care: A Survey-Based Study in a Dutch Hospital
Source: Am J Lifestyle Med. 2025 Oct 25:15598276251384634. Online ahead of print. doi: 10.1177/15598276251384634 (PMC12553549; doi:10.1177/15598276251384634)
Supplement: Supplemental material - Healthcare Professionals’ Perceptions of Lifestyle Medicine in Specialized Care: A Survey-Based Study in a Dutch Hospital [file sj-pdf-1-ajl-10.1177_15598276251384634.pdf]

Healthcare Professionals' Perceptions of Lifestyle Medicine in Specialized Care: A Survey-based Study in a Dutch Hospital

The supplementary materials include 7 supplementary tables.

Legend to supplementary materials

Table S1. Survey questions (English translation).

Table S2. Familiarity with Lifestyle Medicine and its six pillars, split by type of healthcare professional role.

Table S3. Responses to the question: "To what extent do you agree with the following statements?", split by type of healthcare professional role. Statements related to acceptability and appropriateness of Lifestyle Medicine.

Table S4. Responses to the question: "How large do you estimate the proportion of patients in your clinical practice: A) who would benefit from lifestyle support, and B) with whom lifestyle is discussed".

Table S5. Reported Frequency of Lifestyle Discussion Scenarios with Patients.

Table S6. Responses to the question: "To what extent do you agree with the following statements?", split by type of healthcare professional role. Statements related to barriers and facilitators to adoption of Lifestyle Medicine practices.

Table S7. Participants' Preferences for Learning About Lifestyle Medicine.

Table S1: Survey questions (English translation).

Question	***Response Options***
Screening question	
This survey is exclusively intended for healthcare professionals who provide direct patient care to adults within the UMC Utrecht. We request that you only participate if you meet this criterion. Do you meet this criterion?	• Yes<br>• No
Professional background	
Q1[¶]. What type of healthcare professional are you?	• Physician<br>• Nurse<br>• Other
Familiarity with Lifestyle Medicine	
Q2[¶]. Are you familiar with the 6 pillars of lifestyle medicine?	• Yes<br>• No<br>• I am not sure
Informative Text:	
Lifestyle medicine is a discipline within allopathic (western) medicine. It utilizes a range of care models, including health coaching and wellness coaching. It also focuses on developing skills to support behavior change. This includes prescribed lifestyle changes aimed at preventing and addressing the root causes of chronic conditions, and applying lifestyle interventions in curative healthcare as part of the medical treatment of diseases.<br><br>The six pillars of the lifestyle wheel[*] are nutrition, stress management, physical activity, restorative sleep, avoiding risky substances, and social connection. A broader definition of lifestyle medicine includes addressing the upstream social determinants of health (the conditions in which people are born, grow, live, and work).	
Acceptability and Appropriateness	
Q3[†]. To what extent do you agree with the following statements?<br><br>*A)* There should be a stronger focus on lifestyle factors within healthcare.<br>*B)* Investing in lifestyle programs is an efficient way to reduce the pressure on the healthcare system.<br>*C)* Investing in lifestyle programs is an efficient way to manage chronic diseases.<br>*D)* Investing in lifestyle programs is an efficient way to reduce health inequalities.<br>*E)* Implementing a lifestyle program for patients at the UMC Utrecht should be a priority.	• Strongly disagree<br>• Disagree<br>• Neutral<br>• Agree<br>• Strongly agree
OE1[**]. Can you elaborate on your previous answers? Feel free to share additional insights you think are relevant.	Open box.
Adoption – Intention and current practices	
Q4[‡]. Approximately what proportion of your patients do you estimate would benefit from support with lifestyle changes?	• None (0-5%)<br>• Few (6-25%)<br>• Some (26-50%)

	• Most (51-75%)<br><br>Nearly all (> 75%)
Q5[‡]. How large do you estimate the group of patients with whom you discussed lifestyle in the past six months? Discussing lifestyle includes everything from naming lifestyle to discussing it in detail or using techniques such as motivational interviewing.	• None (0-5%)<br>• Few (6-25%)<br>• Some (26-50%)<br>• Most (51-75%)<br>• Nearly all (> 75%)
Q6[§]. How often did the following scenarios occur when you discussed lifestyle with your patients?<br><br>*A)* I mention the importance of lifestyle in general.<br>*B)* I mention the importance of a specific aspect of their lifestyle in relation to the health issue that brought them to my consultation.<br>*C)* I inquire about the patient's lifestyle.<br>*D)* I share lifestyle change success stories from other patients with the same health problems.<br>*E)* I ask questions about the willingness to change lifestyle.<br>*F)* I use health coaching techniques such as motivational interviewing.	• Never to rarely (0-5%)<br>• Occasionally (6-25%)<br>• Regularly (26-50%)<br>• Frequently (51-75%)<br>• (Almost) always (76-100%)
Q7[§]. When you discussed lifestyle with your patients, how often did you discuss the following topics?<br><br>*A)* Diet.<br>*B)* Physical activity.<br>*C)* Sleep.<br>*D)* Social connectivity.<br>*E)* Consumption of risky substances (smoking, alcohol, or drug use).<br>*F)* Stress management.<br>*G)* Cultural aspects and spiritual beliefs.<br>*H)* Patients' support networks.	• Never to rarely (0-5%)<br>• Occasionally (6-25%)<br>• Regularly (26-50%)<br>• Frequently (51-75%)<br>• (Almost) always (76-100%)
Adoption – Barriers and facilitators	
Q8[†]. To what extent do you agree with the following statements?<br><br>*A)* I am confident in my knowledge of the role of lifestyle in the prevention and treatment of diseases.<br>*B)* I am confident in my ability to open a conversation about lifestyle with patients.<br>*C)* I am confident in my ability to promote lifestyle behavior change in patients.<br>*D)* My patients benefit from talking to me about lifestyle during my consultations.<br>*E)* Discussing lifestyle with my patients is part of my job.<br>*F)* I know how to refer my patients who can benefit from lifestyle changes.<br>*G)* Having (more) lifestyle conversations with my patients would increase my workload.<br>*H)* If I would pay more attention to lifestyle with my	• Strongly disagree<br>• Disagree<br>• Neutral<br>• Agree<br>• Strongly agree

patients, I would have a healthier lifestyle myself.	
Q9[¶¶]. What do you consider to be the biggest barrier when discussing lifestyle with your patients? Select all options that apply most to you.	• I lack confidence in my knowledge and/or skills in the field of lifestyle medicine.<br>• I don't think discussing lifestyle is effective.<br>• My patient's socio-economic barriers.<br>• I don't consider it part of my job.<br>• I don't think my patients are interested or motivated enough to discuss lifestyle.<br>• I don't have enough time to discuss lifestyle with my patients.<br>• I don't know how or where to refer my patients to lifestyle programs.<br>• Other.
Q10[‖]. What would help or motivate you to refer more patients to a lifestyle program in your daily clinical practice? Rank in order of relevance.	• The ability to collaborate directly with the program.<br>• Receive a budget or incentive to attend educational activities.<br>• The success stories of patients.<br>• If it is recommended by colleagues.<br>• A clearly structured protocol and information about what the program is about.<br>• A simple and straightforward referral process.<br>• If my manager/department requires this.
Adoption – Interest in LM education	
Q11[¶]. Are you interested in expanding your knowledge in the field of lifestyle medicine?	• Yes<br>• No<br>• Maybe
Q12[¶¶]. What would be your preferred method for learning more about lifestyle medicine and its applicability to your clinical practice? Select the three options that apply most to you.	• E-learning module offered by the hospital.<br>• Guidance from a specialist in this field.<br>• Institutional newsletter containing relevant journal publications in the field of Lifestyle medicine.<br>• Lifestyle Medicine Podcast.<br>• Educational workshops or seminars

	(Online).<br>• Webinars by own Department.<br>• Attending a symposium or conference on Lifestyle Medicine.<br>• Formal educational program (postgraduate courses or certifications).<br>• Free subscription to the "Dutch Association of Lifestyle Medicine" and their sources.<br>• Other
OE2**. Please share any additional thoughts, suggestions, questions, or comments about what is needed for the successful implementation and integration of lifestyle medicine at UMC Utrecht.	Open box.

Notes: Abbreviations: Q = close-ended question; OE = open-ended question.

Q7 and Q8 were not shown to participants who answered "never" in Q6. Q13 was not shown to participants who answered "no" in Q12.

**The 'Lifestyle wheel' is a tool to support the conversation between the healthcare professional and the patient, developed by the the Dutch Association of Lifestyle Medicine 'Arts en Leefstijl': https://www.artsenleefstijl.nl/leefstijlroer.*

*†5-point Likert Scale (1 = completely disagree, 5 = completely agree);; ‡5-point Likert Scale about proportion of patients from clinical practice, ranging from never (0-5%) to almost all patients (>75%); §5-point Likert Scale about frequency of clinical scenarios from never to rarely (0-5%) to almost always (> 75%); ¶Single selection; ¶¶ Multiple selection; ||Graded items in order of relevance (1 = most relevant, 7 = least relevant); **Free-text response.*

Table S2. Distribution of responses to the question "Are you familiar with Lifestyle Medicine and its six pillars?" (Question 2).

	Yes	No	Unsure	Total
**Physicians**	19 (20%)	67 (69%)	11 (11%)	97
**Nurses**	9 (7.5%)	101 (85%)	9 (7.5%)	119
**Others**	15 (17%)	59 (68%)	13 (15%)	87
**Total**	**43 (14%)**	**227 (75%)**	**33 (11%)**	**303 (100%)**

Note: Data are categorized by type of healthcare professional. No statistically significant differences among professional roles were found (Pearson's Chi-squared test, $p > 0.05$).

Table S3. Responses to question 3: "To what extent do you agree with the following statements?" Statements related to acceptability and appropriateness of Lifestyle Medicine.

A) "There should be a stronger focus on lifestyle factors within healthcare".

	Physicians	Nurses	Others	Total
**Strongly Disagree**	1% (1)	0% (0)	0% (0)	0.3% (1)
**Disagree**	3.1% (3)	1.7% (2)	1% (1)	2% (6)
**Neutral**	9.3% (9)	2.5% (3)	7% (6)	6% (18)
**Agree**	38.1% (37)	57.1% (68)	59% (51)	52% (156)
**Strongly Agree**	48.5% (47)	38.7% (46)	33% (29)	40.2% (122)
**Total**	**100% (97)**	**100% (119)**	**100% (87)**	**100% (303)**
**Mean (*SD*)**	**4.3 (0.8)**	**4.3 (0.6)**	**4.2 (0.6)**	**4.3 (0.7)**

B) "Investing in lifestyle programs is an efficient way to reduce the pressure on healthcare".

	Physicians	Nurses	Others	Total
**Strongly Disagree**	1% (1)	0.8% (1)	0% (0)	0.7% (2)
**Disagree**	7.2% (7)	1.7% (2)	2.3% (2)	3.6% (11)
**Neutral**	23.7% (23)	12.6% (15)	9.2% (8)	15.2% (46)
**Agree**	40.2% (39)	53.8% (64)	57.5% (50)	50.5% (153)
**Strongly Agree**	27.8% (27)	31.1% (37)	31% (27)	30% (91)
**Total**	**100% (97)**	**100% (119)**	**100% (87)**	**100% (303)**
**Mean (*SD*)**	**3.9 (0.9)**	**4.1 (0.8)**	**4.2 (0.7)**	**4.1 (0.8)**

C) ***"Investing in lifestyle programs is an efficient way of managing chronic diseases".***

	Physicians	Nurses	Others	Total
**Strongly Disagree**	1% (1)	0% (0)	0% (0)	0.3% (1)
**Disagree**	3.1% (3)	1.7% (2)	1% (1)	2% (6)
**Neutral**	23.7% (23)	18.5% (22)	23% (20)	21.5% (65)
**Agree**	46.4% (45)	50.4% (60)	52% (45)	49.5% (150)
**Strongly Agree**	25.8% (25)	29.4% (35)	24% (21)	26.7% (81)
**Total**	**100% (97)**	**100% (119)**	**100% (87)**	**100% (303)**
**Mean (*SD*)**	**3.9 (0.8)**	**4.1 (0.7)**	**4.0 (0.7)**	**4.0 (0.8)**

D) ***"Investing in lifestyle programs is an efficient way to reduce health inequalities".***

	Physicians	Nurses	Others	Total
**Strongly Disagree**	2% (2)	0% (0)	0% (0)	0.7% (2)
**Disagree**	12.4% (12)	3.4% (4)	6% (5)	6.9% (21)
**Neutral**	20.6% (20)	23.5% (28)	23% (20)	22.4% (68)
**Agree**	42.3% (41)	54.6% (65)	54% (47)	50.5% (153)
**Strongly Agree**	22.7% (22)	18.5% (22)	17% (15)	19.5% (59)

**Total**	**100% (97)**	**100% (119)**	**100% (87)**	**100% (303)**
**Mean (*SD*)**	**3.7 (1.0)**	**3.9 (0.7)**	**3.8 (0.8)**	**3.8 (0.8)**

E) "Implementing a lifestyle program for patients at the UMC Utrecht should be a priority".

	**Physicians**	**Nurses**	**Others**	**Total**
**Strongly Disagree**	7% (7)	1% (1)	0% (0)	3% (8)
**Disagree**	10% (10)	3% (4)	8% (7)	7% (21)
**Neutral**	26% (25)	16% (19)	23% (20)	21% (64)
**Agree**	30% (29)	57% (68)	53% (46)	47% (143)
**Strongly Agree**	27% (26)	23% (27)	16% (14)	22% (67)
**Total**	**100% (97)**	**100% (119)**	**100% (87)**	**100% (303)**
**Mean (*SD*)**	**3.6 (1.2)***	**4.0 (0.8)***	**3.8 (0.8)**	**3.8 (0.8)**

Notes: Participants responded using a 5-point Likert scale (1 = Strongly disagree, 5 = Strongly agree).

Results are displayed using two metrics: (1) the percentage and number of participants selecting each response option, and (2) the mean score with standard deviation for each statement. Data are categorized by type of healthcare professional.

Statistically significant differences in mean scores between nurses and physicians ($p < 0.05$, based on ANOVA with Tukey post hoc test); nurses reported higher agreement than physicians.

Table S4. Responses to questions 4 and 5: "How large do you estimate the proportion of patients in your clinical practice A) who would benefit from lifestyle support, and B) with whom lifestyle is discussed?"

A) "Who would benefit from support with lifestyle behavior change".				
	**Physicians**	**Nurses**	**Others**	**Total**
**None (0-5%)**	2% (2)	2% (3)	5% (4)	3% (9)
**Few (6-25%)**	10% (10)	13% (15)	7% (6)	10% (31)
**Some (26-50%)**	30% (29)	23% (27)	31% (27)	28% (83)
**Most (51-75%)**	43% (42)	50% (60)	40% (35)	45% (137)
**Nearly all (76-100%)**	15% (14)	12% (14)	17% (15)	14% (43)
**Total**	**100% (97)**	**100% (119)**	**100% (87)**	**100% (303)**
B) "With whom you raised or discussed the topic of lifestyle in the past six months"*				
	**Physicians**	**Nurses**	**Others**	**Total**
**None (0-5%)**	7% (7)	22% (26)	28% (24)	19% (57)
**Few (6-25%)**	44% (43)	46% (55)	26% (23)	40% (121)
**Some (26-50%)**	24% (23)	15% (18)	16% (14)	18% (55)
**Most (51-75%)**	11% (11)	10% (12)	18% (16)	13% (39)
**Nearly all (76-100%)**	14% (13)	7% (8)	12% (10)	10% (31)
**Total**	**100% (97)**	**100% (119)**	**100% (87)**	**100% (303)**

Notes: Results are displayed using the percentage and number of participants selecting each option. Data are categorized by type of healthcare professional.

**Statistically significant differences observed, with physicians differing significantly from nurses and other HCPs (based on Pearson's Chi-squared test, $p > 0.05$).*

Table S5. Reported Frequency of Lifestyle Discussion Scenarios with Patients, among those healthcare professionals regularly discussing lifestyle (responses to questions 6 and 7).

A) **Lifestyle Practices/Techniques**	**Never to rarely (0-5%)**	**Occasionally (6-25%)**	**Regularly (26-50%)**	**Frequently (51-75%)**	**Almost always (76-100%)**
**N = 246**					
I mention the importance of lifestyle in general.	4% (9)	13% (31)	26% (65)	52% (129)	5% (12)
I mention the importance of a specific aspect of the patients' lifestyle in relation to the health issue that brought them to my consultation.	4.5% (11)	5.3% (13)	25.6% (63)	46.3% (114)	18.3% (45)
I inquire about the patients' lifestyle.	0.4% (1)	8.5% (21)	24% (59)	51.2% (126)	15.9% (39)
I share lifestyle change success stories from other patients with the same health problems.	28% (69)	30% (74)	26% (63)	14% (34)	2% (6)
I ask questions about the willingness to change lifestyle.	6% (15)	22% (54)	30% (75)	34% (83)	8% (19)
I use health coaching techniques such as motivational interviewing.	16% (40)	27% (67)	25% (61)	26% (63)	6% (15)
B) Lifestyle Topics.	**Never to rarely (0-5%)**	**Occasionally (6-25%)**	**Regularly (26-50%)**	**Frequently (51-75%)**	**Almost always (76-100%)**
**N = 246**					
Pillars of Lifestyle Medicine					
Physical activity.	2% (6)	3% (8)	18% (43)	52% (127)	25% (62)
Diet.	4.1% (10)	7.3% (18)	21.5% (53)	47.6% (117)	19.5% (48)
Consumption of risky substances.	6% (14)	7% (16)	23% (57)	37% (92)	27% (67)
Sleep habits.	13% (32)	17% (41)	28% (69)	32% (79)	10% (25)
Stress management.	15% (38)	24% (58)	32% (78)	23% (57)	6% (15)
Social connectivity.	20% (50)	26% (64)	28% (68)	19% (47)	7% (17)
Contextual factors for behavior change					
Patients' supporting network.	11% (28)	19% (46)	28% (69)	34% (83)	8% (20)
Cultural aspects and spiritual beliefs.	40% (98)	33% (81)	18% (45)	8% (19)	1% (3)

Table S6. Responses to question 8: "To what extent do you agree with the following statements?" Statements related to barriers and facilitators to adoption of Lifestyle Medicine practices.

***A)* “I am confident in my knowledge of the role of lifestyle in preventing and treating disease”**				
	**Physicians**	**Nurses**	**Others**	**Total**
**Strongly Disagree**	1% (1)	0% (0)	0% (0)	0.3% (1)
**Disagree**	9% (9)	12% (14)	9.2% (8)	10.2% (31)
**Neutral**	31% (30)	24% (29)	31% (27)	28.4% (86)
**Agree**	54% (52)	59% (70)	48.3% (42)	54.1% (164)
**Strongly Agree**	5% (5)	5% (6)	11.5% (10)	6.9% (21)
**Total**	**100% (97)**	**100% (119)**	**100% (87)**	**100% (303)**
**Mean (*SD*)**	**3.5 (0.8)**	**3.6 (0.8)**	**3.6 (0.8)**	**3.6 (0.8)**
**B) “I am confident in my ability to open a conversation about lifestyle with patients”**				
	**Physicians**	**Nurses**	**Others**	**Total**
**Strongly Disagree**	1% (1)	0% (0)	0% (0)	0.3% (1)
**Disagree**	12% (12)	9% (11)	5.7% (5)	9.2% (28)
**Neutral**	22% (21)	17% (20)	21.8% (19)	19.8% (60)
**Agree**	56% (54)	65% (77)	59.8% (52)	60.4% (183)
**Strongly Agree**	9% (9)	9% (11)	12.6% (11)	10.2% (31)
**Total**	**100% (97)**	**100% (119)**	**100% (87)**	**100% (303)**
**Mean (*SD*)**	**3.6 (0.9)**	**3.7 (0.8)**	**3.8 (0.7)**	**3.7 (0.8)**
**C) “I am confident in my ability to promote lifestyle behaviour change in patients”**				
	**Physicians**	**Nurses**	**Others**	**Total**
**Strongly Disagree**	2.1% (2)	0.8% (1)	0% (0)	1% (3)
**Disagree**	26.8% (26)	13.4% (16)	12.6% (11)	17% (53)
**Neutral**	38.1% (37)	36.1% (43)	35.6% (31)	37% (111)
**Agree**	27.8% (27)	46.2% (55)	39.1% (34)	40% (120)
**Strongly Agree**	5.2% (5)	3.4% (4)	12.6% (11)	5% (16)
**Total**	**100% (97)**	**100% (119)**	**100% (87)**	**100% (303)**
**Mean (*SD*)**	**3.1 (0.9)***	**3.4 (0.8)***	**3.5 (0.9)***	**3.3 (0.9)**
**D) “(More) lifestyle discussions with my patients would increase my workload”**				
	**Physicians**	**Nurses**	**Others**	**Total**
**Strongly Disagree**	1% (1)	1% (1)	2% (2)	1.3% (4)
**Disagree**	1% (1)	15% (18)	18% (16)	11.6% (35)
**Neutral**	15.5% (15)	24% (29)	39% (34)	25.7% (78)
**Agree**	44.3% (43)	45% (53)	28% (24)	39.6% (120)

**Strongly Agree**	38.1% (37)	15 (18)	13% (11)	21.8% (66)
**Total**	**100% (97)**	**100% (119)**	**100% (87)**	**100% (303)**
**Mean (*SD*)**	**4.2 (0.8)**	**3.6 (1.0)**	**3.3 (1.0)**	**3.8 (0.9)**

E) "Discussing lifestyle with my patients is part of my job"

	Physicians	Nurses	Others	Total
**Strongly Disagree**	4% (4)	4% (5)	9% (8)	5.6% (17)
**Disagree**	6% (6)	19% (23)	18% (16)	14.9% (45)
**Neutral**	18% (17)	24% (28)	15% (13)	19.1% (58)
**Agree**	59% (57)	45% (54)	43% (37)	48.8% (148)
**Strongly Agree**	13% (13)	8% (9)	15% (13)	11.6% (35)
**Total**	**100% (97)**	**100% (119)**	**100% (87)**	**100% (303)**
**Mean (*SD*)**	**3.7 (0.9)***	**3.3 (1.0)***	**3.4 (1.2)**	**3.4 (0.8)**

F) "I know how to refer my patients who could benefit from lifestyle changes"

	Physicians	Nurses	Others	Total
**Strongly Disagree**	10% (10)	6% (7)	9% (8)	8.3% (25)
**Disagree**	34% (33)	41% (49)	29% (25)	35.3% (107)
**Neutral**	26% (25)	25% (30)	26% (23)	25.7% (78)
**Agree**	25% (24)	22% (26)	28% (24)	24.4% (74)
**Strongly Agree**	5% (5)	6% (7)	8% (7)	6.3% (19)
**Total**	**100% (97)**	**100% (119)**	**100% (87)**	**100% (303)**
**Mean (*SD*)**	**2.8 (1.1)**	**2.8 (1.0)**	**3.0 (1.1)**	**2.9 (0.9)**

G) "My patients benefit from talking to me about lifestyle during my consultations"

	Physicians	Nurses	Others	Total
**Strongly Disagree**	1% (1)	3% (4)	3% (3)	3% (8)
**Disagree**	6% (6)	13% (15)	8% (7)	9% (28)
**Neutral**	23% (22)	33% (39)	29% (25)	28% (86)
**Agree**	62% (60)	47% (56)	52% (45)	53% (161)
**Strongly Agree**	8% (8)	4% (5)	8% (7)	7% (20%)
**Total**	**100% (97)**	**100% (119)**	**100% (87%)**	**100% (303)**
**Mean (*SD*)**	**3.7 (0.8)**	**3.4 (0.9)**	**3.5 (0.9)**	**3.5 (0.9)**

H) "If I paid more attention to lifestyle in my patients, I would have a healthier lifestyle myself"

	Physicians	Nurses	Others	Total
**Strongly Disagree**	34% (33)	16% (19)	17.2% (15)	22% (67)
**Disagree**	35% (34)	38.7% (46)	34.5% (30)	36% (110)
**Neutral**	21% (20)	32.8% (39)	29.9% (26)	28% (85)
**Agree**	8% (8)	10.9% (13)	17.2% (15)	12% (36)

**Strongly Agree**	2% (2)	1.7% (2)	1.1% (1)	2% (5)
**Total**	**100% (97)**	**100% (119)**	**100% (87)**	**100% (303)**
**Mean (*SD*)**	**2.1 (1.0)***	**2.4 (0.9)***	**2.5 (1.0)***	**2.3 (1.0)**

Notes: Participants responded using a 5-point Likert scale (1 = Strongly disagree, 5 = Strongly agree).

Results are displayed using two metrics: (1) the percentage and number of participants selecting each response option, and (2) the mean score with standard deviation for each statement. Data are categorized by type of healthcare professional.

Statistically significant differences in mean scores among professional roles ($p < 0.05$, based on ANOVA with Tukey post hoc test).

Table S7. Participants' Preferences for Learning About Lifestyle Medicine (Question 12).

**A) Are you interested in expanding your knowledge in the field of Lifestyle Medicine?** Percentage and number of participants selecting each option, split by type of healthcare professional.				
	**Yes**	**Maybe**	**No**	**Total**
**Physicians**	40% (39)	35% (34)	25% (24)	100% (97)
**Nurses**	56% (67)	31% (37)	13% (15)	100% (119)
**Others**	56% (49)	28% (24)	16% (14)	100% (87)
**Total**	**155 (51%)**	**53 (18%)**	**95 (31%)**	**303 (100%)**

**B) Number of times each learning method was selected, among those interested in learning more (n = 250).** Up to 3 choices per participant.	
**Learning method**	**Frequency of option selection**
E-learning module offered by the institution (UMC Utrecht).	156
Educational workshops or seminars delivered by own department.	139
Symposium or conference on Lifestyle Medicine.	109
Online webinars.	70
Mentorship by a specialist in this field.	60
Formal educational program (postgraduate courses or certifications in Lifestyle Medicine).	58
Lifestyle Medicine podcast.	52
Institutional newsletter sharing relevant journal publications in the field of Lifestyle Medicine.	49
Free subscription to resources of the national Lifestyle Medicine organization.	46
Others:	
- *Advertisement and practical guidelines of already available initiatives.*	2
- *Request for not receiving more mandatory training.*	2
- *Learning from patients' associations.*	1
- *Sharing best practices.*	1
- *Workshop on motivational interviewing.*	1
- *Learning with the example (request for improving healthy catering in departments).*	1
